# Prevalence of hypertension and its treatment among adults presenting to primary health clinics in rural Zambia: analysis of an observational database

**DOI:** 10.1186/s12889-015-2258-4

**Published:** 2015-09-21

**Authors:** Lily D. Yan, Benjamin H. Chi, Ntazana Sindano, Samuel Bosomprah, Jeffrey SA Stringer, Roma Chilengi

**Affiliations:** Primary Care and Health Systems Department, Center for Infectious Disease Research Zambia, Lusaka, Zambia; Department of Obstetrics and Gynecology, University of North Carolina School of Medicine, Chapel Hill, North Carolina USA; Stanford University School of Medicine, Stanford, California USA; Department of Biostatistics, School of Public Health, University of Ghana, Accra, Ghana

**Keywords:** Hypertension, Prevalence, Antihypertensive agents, Primary health care, Zambia

## Abstract

**Background:**

Hypertension constitutes a growing burden of illness in developing countries like Zambia. Adequately screening and treating hypertension could greatly reduce the complications of stroke and coronary disease. Our objective was to determine the prevalence of hypertension and identify current treatment practices among adult patients presenting for routine care to rural health facilities in the Better Health Outcomes through Mentoring and Assessments (BHOMA) project.

**Methods:**

We conducted a retrospective analysis of routinely collected clinical data from 46 rural government clinics in Zambia. Our analysis cohort comprised patients ≥ 25 years with recorded blood pressure measurements, who sought care at primary health centers. Consistent with prior research, in our primary analysis, we only included data from first visits. Hypertension was defined as a systolic blood pressure ≥140 mmHg, or diastolic blood pressure ≥90 mmHg, or reported use of antihypertensive medication. A sensitivity analysis was performed using median blood pressure for individuals with multiple visits.

**Results and Discussion:**

From January 2011 to December 2014, 116,130 first visits by adult patients met eligibility criteria. The crude prevalence of hypertension by onsite measurement or reported use of antihypertensive medication was 23.1 % [95 % CI: 22.8-23.3] (23.6 % in females, 22.3 % in males). The age standardized prevalence of hypertension across participating sites was 28.0 % [95 % CI: 27.7-28.3] (29.7 % in females, 25.8 % in males). Sensitivity analysis revealed a similar prevalence using data from all visits. Only 5.6 % of patients had a diagnosis of hypertension documented in their medical record. Among patients with hypertension, only 18.0 % had any antihypertensive drug prescribed, with nifedipine (8.9 %), furosemide (8.3 %), and propranolol (2.4 %) as the most common.

**Conclusions:**

Age standardized prevalence of hypertension in rural primary health clinics in Zambia was high compared to other studies in rural Africa; however, we diagnosed hypertension with only one measurement and this may have biased our findings. Future efforts to improve hypertension control should focus on population preventive care and primary healthcare provider education on individual management.

**Electronic supplementary material:**

The online version of this article (doi:10.1186/s12889-015-2258-4) contains supplementary material, which is available to authorized users.

## Background

Hypertension and its long term consequences constitute a growing burden in developing countries, with as much as 75 % of all affected patients now living in low- and middle- income countries (LMIC) [1–5]. Defined by the World Health Organization as a blood pressure measurement ≥140/90 mmHg, hypertension is a major known risk factor for chronic conditions such as cardiovascular disease, hemorrhagic stroke, and renal failure [1, 6–9]. According to the 2012 WHO Global Health Observatory, 46 % of deaths in LMIC are attributable to cardiovascular disease (CVD) [10]. Notably, CVD deaths occur in sub-Saharan Africa at a younger mean age (64.9 years) than anywhere else in the world [7].

In Zambia, non-communicable diseases (NCDs) account for up to a quarter of all deaths, with about half of these due to CVDs [11]. As life expectancy increases, this burden is expected to rise as well. There is a paucity of epidemiologic data on hypertension prevalence and treatment in Zambia [12, 13]. Like the vast majority of studies in sub-Saharan Africa, past studies in Zambia have used cross-sectional community survey designs with a single blood pressure (BP) measurement to define hypertension [12–15]. To date, there are no studies examining the profile of hypertension patients who actually present to primary health clinics. Similarly, while control of hypertension with appropriate medication can greatly reduce complications like stroke and other cardiovascular events [16], there are very few studies that have examined and reported treatment practices.

To address these gaps, we sought to determine the age standardized prevalence and existing treatment practices of hypertension in 46 Zambian rural primary health care centers participating in the Better Health Outcomes through Mentoring and Assessment (BHOMA) intervention [17].

## Methods

The BHOMA project is a five-year, randomized stepped-wedge trial of improved clinical service delivery underway in 46 rural clinics in Chongwe, Kafue, and Luangwa Districts. The public sector lacks robust healthcare infrastructure and suffers from a severe human resource shortage, with only 0.7 physicians and 7.8 nurses per 10,000 people [18]. A number of our selected rural clinics are staffed by one nurse or clinical officer, and frequently suffer from equipment shortage. BHOMA has five principal objectives: 1) creating standardized protocols and forms for common primary care visits, 2) ensuring clinics are properly equipped with essential diagnostic and management tools, 3) on site electronic medical records (EMR), 4) improving key indicators with ongoing mentoring, and 5) increasing community engagement with community health workers and traditional birth attendants [17]. This study is an analysis of the observational EMR database.

### Participants and data collection

First primary care visits by adults ≥25 years with a blood pressure measurement were included, to collapse the longitudinal database into a cross-sectional sample. Age bands from 0–25 years were dropped in calculations consistent with prior studies [9]. BHOMA intervention began in Aug 2010 in six pilot primary health facilities, and Apr 2011 in 40 primary health facilities, with current ongoing follow up. The EMR database was operational starting Jan 2011 and captures data from all clinic visits by children and adults; thus all data from Jan 2011 to Dec 2014 was included in this analysis. Demographic, medical assessment and treatment data were all collected as part of routine care through the standardized forms, and entered weekly into the EMR via computer interface to a central, backed up server. All vital signs, including blood pressure, were taken during visits by either the government clinician, or clinic support workers trained and provided by BHOMA. Clinic support workers were trained to take blood pressure using an appropriate sphygmomanometer with a cuff of at least 40 % the arm circumference in width, with the patient sitting in an upright position after a period of rest. At BHOMA’s inception, all study clinics were provided with such digital blood pressure monitors. More details on program implementation and data capture methods are described elsewhere [17, 19].

### Analysis

The primary outcome was hypertension by measurement, defined as either measured systolic blood pressure (SBP) ≥140 mmHg, diastolic blood pressure (DBP) ≥90 mmHg, or prescription/reported use of antihypertensive medication.

To provide insight into the discrepancy between actual measured hypertension burden and recognized hypertension burden by healthcare workers, a person was classified as having hypertension by diagnosis if a healthcare worker formally recorded a diagnosis of hypertension in that patient’s medical record, or prescribed an antihypertensive agent. The presence of diabetes mellitus, heart failure, and stroke were extracted from the medical record as well. The prevalence of hypertension was calculated as a proportion with 95 % confidence intervals.

As a secondary outcome to analyze treatment practices, prescribed antihypertensive medications were examined for people with hypertension by measurement. We calculated the proportion of people that had a prescribed drug at their first visit. Analysis was also performed for the subset of people who had uncontrolled hypertension, which we defined as people prescribed antihypertensive medication with an elevated BP measurement. Controlled hypertension was defined as people on medication with a normal BP measurement. Bivariate analysis of independent variables such as demographic and treatment characteristics, for the outcome of uncontrolled versus controlled hypertension, was completed using a Chi square test with a p value of <0.05 as statistically significant.

To account for the confounding effect of age, age standardized prevalence of hypertension was estimated using direct standardization methods. That is, observed prevalence of hypertension for each age strata were multiplied by the WHO World Standard’s weights for each stratum, and summed together to calculate the age standardized prevalence of hypertension [20].

In order to understand the effect of repeat visits on calculation of hypertension prevalence, a sensitivity analysis was performed using data from all visits by adults ≥25 years, rather than the first visit alone, to calculate the age standardized prevalence. An individual was classified as hypertensive if the median of all BP measurements across visits was either SBP ≥140 or DBP ≥90, or if they were ever prescribed antihypertensive medication.

Lastly, multivariable logistic regressions were built for two outcomes: 1) hypertension among all patients, and 2) hypertension control among patients on antihypertensive medications (as defined above). The predictors age, gender, and body mass index (BMI) were included as categorical independent variables; smoking was not included as this data was not collected. Visits missing any of the independent variables were dropped. First, only main effects of the independent variables were included. Second, comparisons of the predicted and actual crude prevalence of hypertension within each strata were compared to determine if there was a possibility of interaction between two variables. Third, final models including main effects and all interactions were built for independent variables, as the effect of age and BMI were hypothesized to be dependent on the patient gender. Odds ratios (OR) from these final models are presented for the predictor variables, and the log of the ratio of odds ratios are presented for the interaction terms. All analyses were performed in Stata/SE 13.1 (StataCorp, College Station, TX).

### Ethics statement

This study was approved by the Biomedical Research Ethics Committee at the University of Zambia and the Biomedical Institutional Review Board at the University of North Carolina at Chapel Hill. For individual patients presenting to BHOMA clinics, the requirement for informed consent was waived for use of these de-identified routinely collected program data by both regulatory bodies. The authors confirm that all ongoing and related trials for this intervention are registered. BHOMA is registered at ClinicalTrials.gov, Identifier: NCT01942278.

## Results

**Fig. 1:**
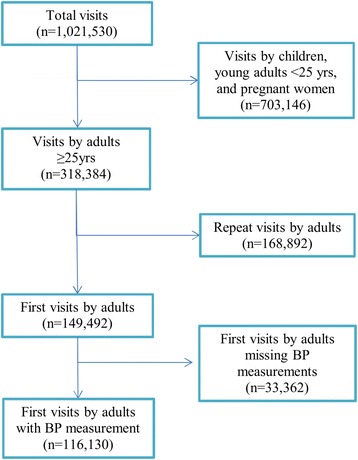
Study Cohort

Between January 2011 and December 2014, a total of 1,021,530 visits were documented at 46 participating BHOMA health facilities. Of these, 318,384 (31.2 %) were primary care visits by adults ≥25 years. After excluding repeat visits and first visits without a BP measurement, a total of 116,130 first visits by adults were left (median number of visits: 1; interquartile range: 1,2). This comprised our analysis cohort (Fig. 1). A total of 48,843 (42.1 %) adults in the cohort made more than one visit.

Fifty seven percent of patients were female, with an overall mean age of 40 years dominated by younger participants under 45 (69.7 %). Most patients (71.6 %) had a body mass index (BMI) <25 kg/m^2^ (Table 1). Our adult study population was significantly older (mean 40 years, median 36 years) compared to the overall population (mean 20 years, median 18 years) that presented to BHOMA facilities in the study timeframe.

**Table 1:** Prevalence of hypertension in 116,130 patients presenting to rural health facilities by background characteristics, Zambia

Characteristic (unit)	Age, mean (SD)	Number of Patients, n (% of total)	Crude prevalence of hypertension, n (% of row)	Age standardized prevalence of hypertension, % [95 % CI^a^]
Age (years)				--
25-34	--	46,547 (40.1)	6497 (14.0)	--
35-44	--	34,406 (29.6)	6761 (19.7)	--
≥45	--	35,177 (30.3)	13,537 (38.5)	--
Sex				
Females	40 (13.9)	66,138 (56.9)	15,635 (23.6)	29.7 [29.3, 30.0]
Males	41 (13.9)	49,992 (43.1)	11,156 (22.3)	25.8 [25.4, 26.2]
BMI (kg/m^2^)				
<18.5	43 (15.6)	16,820 (14.5)	2812 (16.7)	18.3 [17.6, 18.9]
18.5-24.9	39 (13.5)	66,362 (57.1)	13,299 (20.0)	25.4 [25.0, 25.7]
25.0-29.9	42 (13.4)	15,964 (13.7)	4925 (30.8)	36.0 [35.2, 36.8]
≥30	43 (12.8)	7377 (6.4)	3152 (42.7)	46.6 [45.4, 47.7]
Total	40 (13.9)	116,130	26,791 (23.1)	28.0 [27.7, 28.3]

^a^CI confidence interval

### Hypertension prevalence

The crude prevalence of hypertension by measurement was estimated as 23.1 % [95 % CI: 22.8-23.3]. In contrast, the crude prevalence of hypertension by diagnosis was 5.6 % [95 % CI: 5.4-5.7]. On average, the age standardized prevalence of hypertension by measurement was 28.0 % [95 % CI: 27.7-28.3]. When we compared crude versus age standardized estimates, age standardized prevalence was consistently higher across all categories (Table 1). The sensitivity analysis performed using data from all visits by adults ≥25 years, rather than only the first visit, resulted in a slightly lower age standardized prevalence of 26.7 % [95 % CI: 26.4-27.0].

**Fig. 2:**
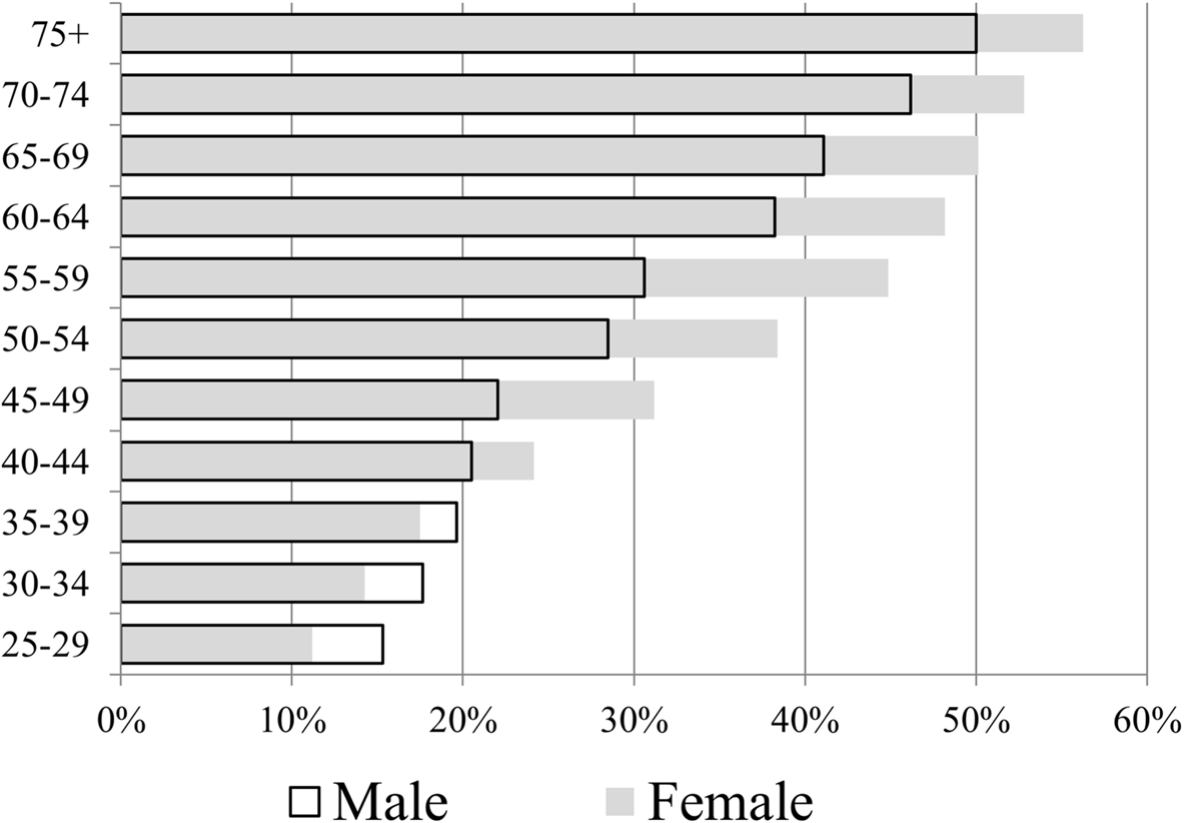
Prevalence of hypertension by age group and sex. Hypertension by measurement. Females represented by gray solid bars, males represented by black outline bars

Figure 2 presents the crude prevalence of hypertension by age group and gender. Although males had a higher prevalence of hypertension compared to females at lower age strata, by the age band 40–44, this trend reversed. For hypertension by diagnosis, females had a higher prevalence than males across all age bands.

Documented comorbidities, such as diabetes mellitus type II, or expected complications of chronic hypertension such as congestive heart failure and stroke, were extremely low at less than 1 % of people each.

In the logistic regression, increases in age or BMI category were associated with stepwise increases in the OR of hypertension (Additional file 1: Table S1). Male gender was found to have a 1.54 OR [95 % CI: 1.39-1.69] of hypertension compared to females. Interestingly, the interaction terms for age and gender revealed that although males had a higher OR than females at younger age categories (25–34 years) for hypertension, this effect reduced with age. For age band 35–44 years, the log ratio of OR was 0.78 [95 % CI: 0.72-0.85] compared to the younger age band, while for age ≥45 years the log ratio of OR was 0.51 [95 % CI: 0.48-0.55], suggesting females are more likely than males to have hypertension in this older range. Interaction terms for age and BMI, or BMI and gender, were not statistically significant.

### Hypertension treatment

Antihypertensive medications prescribed to patients at the BHOMA primary health centers are listed in Table 2. Out of 26,791 people who had hypertension by measurement, 18.0 % were prescribed at least one antihypertensive drug. The most commonly prescribed medications include nifedipine (8.9 %) and furosemide (8.3 %).

**Table 2:** Antihypertensive medications prescribed for people with hypertension in rural health facilities, Zambia

Antihypertensive Medication	Category	People with hypertension (*n* = 26,791)
		n (%)
atenelol	beta blocker	477 (1.8)
enalapril	angiotensin converting enzyme inhibitor	33 (0.12)
furosemide	loop diuretic	2232 (8.3)
hydralazine	vasodilator	51 (0.19)
hydrochlorothiazide amiloride	thiazide and potassium-sparing diuretic	41 (0.15)
methyldopa	alpha2 agonist	222 (0.83)
nifedipine	calcium channel blocker	2394 (8.9)
propranolol	beta blocker	656 (2.4)
Any antihypertensive		4833 (18.0)

When we examined blood pressure measurements among those reportedly on antihypertensive medication, we found that nearly 90 % remained hypertensive by measurement (Table 3). Individuals with uncontrolled hypertension were more likely to be female, older, and have a higher BMI when compared to those who were normotensive on medication. The proportion of individuals with uncontrolled hypertension who had a recorded medical history of high blood pressure was almost twice the proportion of individuals with controlled blood pressure and a recorded history (40.2 % vs 20.7 %, *p* < 0.0001). Antihypertensive drug prescription was not significantly different across most drug types in the uncontrolled compared to the controlled groups, except for nifedipine (52.0 % vs 27.4 %, *p* < 0.0001) and propranolol (12.9 % vs 19.5 %, *p* = 0.0001).

**Table 3:** Differences in characteristics of patients on antihypertensive medications, by hypertension control status

Characteristic (unit)	Uncontrolled Hypertension (*n* = 4340)	Controlled Hypertension (*n* = 493)	p value
	*n* (%)		
Female	3175 (73.2)	339 (68.8)	0.038
Age (years)			<0.0001
25–34	353 (8.1)	113 (22.9)	
35–44	786 (18.1)	103 (20.9)	
≥45	3201 (73.8)	277 (56.2)	
BMI (kg/m^2^)			<0.0001
<18.5	328 (7.6)	47 (9.5)	
18.5-24.9	1634 (37.6)	220 (44.6)	
25.0-29.9	1000 (23.0)	104 (21.1)	
≥30	831 (19.1)	59 (12.0)	
History of hypertension	1747(40.2)	102 (20.7)	<0.0001
Treatments			
atenelol	439 (10.1)	38 (7.7)	0.09
enalapril	28 (0.65)	5 (1.0)	0.35
furosemide	2007 (46.2)	225 (45.6)	0.80
hydralazine	45 (1.0)	6 (1.2)	0.71
hydrochlorothiazide amiloride	34 (0.78)	7 (1.4)	0.14
methyldopa	205 (4.7)	17 (3.4)	0.20
nifedipine	2259 (52.0)	135 (27.4)	<0.0001
propranolol	560 (12.9)	96 (19.5)	0.0001

Controlled hypertension: on antihypertensive medications with a normal blood pressure measurement (<140/90). Uncontrolled hypertension: on antihypertensive medications without a normal blood pressure measurement

In the logistic regression, increases in age and BMI category were associated with a trend of decreased OR of hypertension control, although most ORs were not statistically significant (Additional file 2: Table S2). Age ≥45 years was associated with a 0.25 OR [95 % CI: 0.095-0.67] of control compared with age 25–34 years, while BMI ≥30 kg/m^2^ was associated with a 0.20 OR [95 % CI: 0.048-0.81] compared with BMI <18.5 kg/m^2^. Male gender was not associated with an increased OR of control compared to females [95 % CI 0.67-3.45]. None of the interaction terms were statistically significant.

## Discussion

In our rural primary care setting, similar to other studies in the region, we observed a high prevalence of hypertension among adults seeking care. Increasing age and BMI were associated with increased OR of hypertension. Male gender was associated with increased OR only in the youngest age band, with a lower OR in older age bands compared to females. Treatment coverage was low with only 18 % of patients with hypertension prescribed medication. Individuals with uncontrolled hypertension were more likely to be female, had higher BMIs, and interestingly more likely to be prescribed nifedipine compared to those with controlled hypertension. However, in multivariable regression, only the highest age category ≥45 years and BMI category ≥30 kg/m^2^ were associated with a statistically significant decreased OR of hypertension control.

This study had several limitations. First, external validity is a concern since our study sampled individuals who sought healthcare at primary care facilities, rather than the population as a whole. Consequently, certain results such as the age standardized prevalence of hypertension may be lower than a community age standardized prevalence. Second, only data from the first visit was used from the longitudinal dataset to arrive at prevalence. Similar to other cross sectional studies, BP measurements from one visit may be spuriously elevated, leading to an overestimation. This trend was evident when we considered the median BP over all visits; in that sensitivity analysis, the overall age-standardized prevalence of hypertension was slightly lower compared to first-visit measurements only. Third, the specific indication for a prescribed drug was not known—for example, we could not confirm from the data whether a loop diuretic was prescribed for heart failure or hypertension. Fourth, key independent variables for inclusion in the regression models (smoking status, family history of hypertension, diet, exercise) were not collected as part of the study. These were balanced against the study’s strengths. Data collected at health facilities represented how closely health centers put theoretical guidelines into practice, and are more relevant for informing interventions integrated into existing infrastructure. In addition, calculation of age standardized prevalence allowed comparison to other prevalences standardized to the WHO World Population.

Within Zambia, our crude prevalence of 23.1 % was lower than the crude prevalence found in previous studies in Lusaka urban district of 34.8 % (38.0 % male, 33.3 % female), but closer to the proportions found in rural Kasama district of 30.3 % (31.3 % male, 29.5 % female), and rural Kaoma district of 25.8 % (27.5 % male, 24.6 % female) [12, 13]. The difference between Lusaka urban district and our data was likely due to our rural sample, which presumably has fewer dietary and lifestyle risk factors for developing high blood pressure than urban samples [1, 15]. The prevalence of hypertension in sub-Saharan Africa has ranged from 10–55 % [14, 15]. Our crude prevalence was slightly lower than the urban areas in neighboring countries Zimbabwe (33.3 %) and South Africa (25.0 %), but higher than rural areas in South Africa (10.5 %) [1, 21]. Differences in sampling, or underlying population age and gender distributions, may have contributed to the wide range of reported crude prevalences. Our age standardized prevalence of 28.0 % was similar to the age standardized levels reported by the Millenium Villages in rural Malawi, Rwanda, and Tanzania (22.8 % hypertension) [22].

Similar to other studies in rural sub-Saharan Africa, our proportions for hypertension by measurement were higher in females than males—especially in older age bands. A meta-analysis showed 8 out of 13 total rural African communities found a higher hypertension prevalence in females compared to males [14]. The location of the effect in older age bands, compared to the younger groups, is consistent with research in other world regions [23, 24]. It is thought that older, postmenopausal women lose the protective role of ovarian estrogen on vasodilation and decreased sympathetic activity, thereby resulting in an elevated BP [25]. Female predominance was also more apparent in the hypertension by diagnosis, which is consistent with prior research demonstrating females were more likely to have been detected, to be on treatment, and have hypertension controlled compared to males [14]. Females have more touch points with the healthcare system through antenatal care visits and have better health seeking behavior, meaning they may present more frequently to health centers and be more likely than males to be detected [14, 26, 27]. Indeed, in BHOMA the majority of patients were females (57 % of study cohort vs 51 % of population) [28].

Another major finding in our study was the under- and improper treatment of hypertension. Zambian national guidelines, consistent with the Eighth Joint National Committee criteria, state that after unsuccessful lifestyle modification, antihypertensive treatment should start with one agent (hydrochlorothiazide-amiloride or a calcium channel blocker), with increasing doses or combinations if BP remains elevated [29, 30]. Almost all classes of antihypertensive drugs are on the essential medicines list and provided free in Zambian government clinics, including thiazides, calcium channel blockers, and ACE inhibitors. A major contributor to under-treatment seemed to be under-diagnosis; another contributing factor may have been healthcare worker discomfort with chronic diseases, as 50.7 % of Zambian government health workers feel a lack of expertise in managing hypertension [31]. While under-treatment certainly contributes to persistent hypertension, even the vast majority of treated patients had uncontrolled hypertension. Possible reasons include lack of awareness in the general population about dietary salt contributions to elevated BP, poor patient adherence or clinic attendance for refills, and improper treatment choices by clinic staff. Furosemide was commonly used (in 8 % of hypertensive patients), which is only recommended in the presence of renal or cardiac failure [32]. Given the extremely low numbers of documented cardiac failure in our sample, it is unlikely that patients were evaluated for these conditions at the primary care level, and thus unlikely to be on furosemide for correct indications. Improper treatment is likely tied to many factors, including medication stock outs and lack of training in best practices.

Our study showed the high prevalence of hypertension in Zambian primary care clinics and the need for early intervention. Given limited resources, interventions should focus on population-level and individual-level public health approaches in parallel. Passing national legislation on hypertension risk factors (tobacco, alcohol, poor diet) may have a cost effective yet considerable public health impact. Modeling in LMIC shows that a 33 % tax on tobacco could result in only US$ 3–42 spent per DALY averted from cardiovascular disease due to decreased smoking [33]. At the individual level, emphasis must be placed on identifying those at highest risk and ensuring proper treatment. The WHO and International Society of Hypertension have developed low resource wall charts of the Framingham cardiovascular risk calculator, requiring only gender, SBP, smoking status, and diabetes mellitus type II status [34]. Treating only patients with a total cardiovascular risk of >15 % in primary prevention can be cost effective at US 790-930 per DALY averted, and is cost saving for secondary prevention [33]. Our study also demonstrated individuals were being treated with the ineffective drug furosemide, suggesting the need for broader health systems strengthening in prescribing practices and drug stock outs. There remain many unanswered questions about hypertension in Zambia, including reasons for the poor screening and treatment in the BHOMA clinics (despite its objective to improve quality of care through structured protocols), treatment practices at higher hospital levels of care, and evaluation of NCD training and practices. Future studies will need to explore reasons for hypertension under-treatment to develop sustainable interventions for primary and secondary prevention of cardiovascular disease.

## Conclusions

In summary, in our rural Zambian primary care setting, the age standardized prevalence of hypertension was high at more than one-quarter of adults aged ≥25 years. Consistent with other studies, females were noted to have a higher prevalence than males especially at older ages, likely due to greater engagement with the health system and menopausal status. Treatment coverage was low, with evidence of improper prescriptions for those who were treated. To prevent progression of hypertension to cardiovascular mortality, future interventions should implement dual population preventive care and high risk individual strategies.

## Additional files

Additional file 1: Table S1. Multivariate logistic regression for predictors of hypertension among all patients, *n* = 106,523. File format: .xlsx (XLSX 11 kb) Additional file 2: Table S2. Multivariate logistic regression for predictors of hypertension control among patients taking antihypertensive medication, *n* = 4223. File format: .xlsx (XLSX 10 kb)

## Abbreviations

BHOMA: Better Health Outcomes through Mentoring and Assessment
BP: Blood pressure
CVD: Cardiovascular disease
DBP: Diastolic blood pressure
EMR: Electronic medical records
LMIC: Low- and middle-income countries
NCD: Non-communicable diseases
OR: Odds Ratio
SBP: Systolic blood pressure

## References

[1] Ibrahim MM, Damasceno A (2012). Hypertension in developing countries. Lancet.

[2] Hunter DJ, Reddy KS (2013). Noncommunicable diseases. N Engl J Med.

[3] Nascimento BR, Brant LC, Moraes DN, Ribeiro AL (2014). Global health and cardiovascular disease. Heart.

[4] Yusuf S, Reddy S, Ôunpuu S, Anand S (2001). Global burden of cardiovascular diseases part I: general considerations, the epidemiologic transition, risk factors, and impact of urbanization. Circulation.

[5] WHO (2002). The world health report 2002: reducing risks, promoting healthy life.

[6] Yusuf S, Hawken S, Ôunpuu S, Dans T, Avezum A, Lanas F (2004). Effect of potentially modifiable risk factors associated with myocardial infarction in 52 countries (the INTERHEART study): case–control study. Lancet.

[7] Moran A, Forouzanfar M, Sampson U, Chugh S, Feigin V, Mensah G (2013). The epidemiology of cardiovascular diseases in sub-Saharan Africa: the Global Burden of Diseases, Injuries and Risk Factors 2010 Study. Prog Cardiovasc Dis.

[8] Khatibzadeh S, Farzadfar F, Oliver J, Ezzati M, Moran A (2013). Worldwide risk factors for heart failure: a systematic review and pooled analysis. Int J Cardiol.

[9] WHO (2008). WHO STEPS surveillance manual: The WHO STEPwise approach to chronic disease risk factor surveillance.

[10] WHO Global Health Observatory: Noncommunicable Diseases [database on the Internet]. WHO. 2012. Available from: http://www.who.int/gho/ncd/en/. Accessed: April 25 2015

[11] WHO (2014). Noncommunicable Diseases Country Profiles: Zambia.

[12] Mulenga D, Siziya S, Rudatsikira E, Mukonka VM, Babaniyi O, Songolo P (2013). District specific correlates for hypertension in Kaoma and Kasama rural districts of Zambia. Rural Remote Health.

[13] Goma FM, Nzala SH, Babaniyi O, Songolo P, Zyaambo C, Rudatsikira E (2011). Prevalence of hypertension and its correlates in Lusaka urban district of Zambia: a population based survey. Int Arch Med.

[14] Addo J, Smeeth L, Leon DA (2007). Hypertension in sub-saharan Africa: a systematic review. Hypertension.

[15] Kayima J, Wanyenze RK, Katamba A, Leontsini E, Nuwaha F (2013). Hypertension awareness, treatment and control in Africa: a systematic review. BMC Cardiovasc Disord.

[16] Blood Pressure Lowering Treatment Trialists’ Collaboration (2000). Effects of ACE inhibitors, calcium antagonists, and other blood-pressure-lowering drugs: results of prospectively designed overviews of randomised trials. Lancet.

[17] Stringer J, Chisembele-Taylor A, Chibwesha C, Chi H, Ayles H, Manda H (2013). Protocol-driven primary care and community linkages to improve population health in rural Zambia: the Better Health Outcomes through Mentoring and Assessment (BHOMA) project. BMC Health Serv Res.

[18] WHO (2014). Zambia: Country Health Profile.

[19] Schuttner L, Sindano N, Theis M, Zue C, Joseph J, Chilengi R (2014). A Mobile Phone-Based, Community Health Worker Program for Referral, Follow-Up, and Service Outreach in Rural Zambia: Outcomes and Overview. Telemed J E Health.

[20] Ahmad O, Boschi-Pinto C, Lopez A, Murray C, Lozano R, Inoue M (2011). Age Standardization of Rates: A New WHO Standard.

[21] Matenga JA, Allain TJ, Wilson AO, Adamchak DJ, Senzanje B, Mushangi E (1997). Hypertension management in Zimbabwe--awareness, treatment and blood pressure control. A community-based study. S Afr Med J.

[22] de Ramirez SS, Enquobahrie D, Nyadzi G, Mjungu D, Magombo F, Ramirez M (2010). Prevalence and correlates of hypertension: a cross-sectional study among rural populations in sub-Saharan Africa. J Hum Hypertens.

[23] Wang H, Zhang X, Zhang J, He Q, Hu R, Wang L (2013). Factors associated with prevalence, awareness, treatment and control of hypertension among adults in Southern China: a community-based, cross-sectional survey. PLoS One.

[24] Meng X-J, Dong G-H, Wang D, Liu M-M, Lin Q, Tian S (2011). Prevalence, awareness, treatment, control, and risk factors associated with hypertension in urban adults from 33 communities of China: the CHPSNE study. J Hypertens.

[25] Ashraf MS, Vongpatanasin W (2006). Estrogen and hypertension. Curr Hypertens Rep.

[26] Mufunda E, Albin B, Hjelm K (2012). Differences in health and illness beliefs in Zimbabwean men and women with diabetes. Open J Nurs.

[27] Hjelm K, Nambozi G (2008). Beliefs about health and illness: a comparison between Ugandan men and women living with diabetes mellitus. Int Nurs Rev.

[28] Central Statistical Office (CSO), Ministry of Health (MOH), Tropical Diseases Research Centre (TDRC), University of Zambia, and Macro International Inc (2009). Zambia Demographic and Health Survey 2007.

[29] Ministry of Health Zambia National Formulary Committee (2008). Standard Treatment Guidelines, Essential Medicines List, Essential Laboratory Supplies for Zambia.

[30] James PA, Oparil S, Carter BL, Cushman WC, Dennison-Himmelfarb C, Handler J (2014). 2014 evidence-based guideline for the management of high blood pressure in adults: Report from the panel members appointed to the eighth joint national committee (JNC 8). JAMA.

[31] Ministry of Health Zambia (2010). National Health Strategic Plan 2011–2015.

[32] Weber MA, Schiffrin EL, White WB, Mann S, Lindholm LH, Kenerson JG (2014). Clinical practice guidelines for the management of hypertension in the community: a statement by the American Society of Hypertension and the International Society of Hypertension. J Hypertens.

[33] Gaziano TA (2007). Reducing the growing burden of cardiovascular disease in the developing world. Health Aff (Millwood).

[34] Mendis S, Lindholm LH, Mancia G, Whitworth J, Alderman M, Lim S (2007). World Health Organization (WHO) and International Society of Hypertension (ISH) risk prediction charts: assessment of cardiovascular risk for prevention and control of cardiovascular disease in low and middle-income countries. J Hypertens.

